# Planning and communicating prototype tests for the Nano Membrane Toilet: A critical review and proposed visual tool

**DOI:** 10.12688/gatesopenres.13057.2

**Published:** 2019-11-12

**Authors:** Jan Hennigs, Alison Parker, Matt Collins, Ying Jiang, Athanasios Kolios, Ewan McAdam, Leon Williams, Sean Tyrrel

**Affiliations:** 1 Water Science Institute, Cranfield University, Cranfield, MK430AL, UK; 2 Freeform Design & Innovation Ltd., Flitwick, UK; 3 University of Strathclyde, Glasgow, UK

**Keywords:** prototyping, reinvent the toilet, testing, waterless sanitation

## Abstract

Urban sanitation in growing cities of the Global South presents particular challenges, like the speed of their growth, the high population density, and, often, the lack of existing wastewater infrastructure. This led to the Bill & Melinda Gates Foundation’s Reinvent The Toilet Challenge, a call to develop novel, non-sewered sanitation technologies, which sparked the development of various inventions, like the Nano Membrane Toilet. Complex technologies like this entail an extensive product development process, including various iterations of prototype tests. While there is an abundance of literature discussing how to build prototypes, and the optimal number of tests, there has been little focus on how to plan, communicate, and conduct tests, especially in a product development endeavour of this complexity. Multiple aspects of testing prototypes are reviewed. A visual test planning tool is proposed that encompasses the entire product development process and can be used to plan and communicate prototype tests for the Nano Membrane Toilet to ultimately achieve compliance with international standards.

## Abbreviations

ALT – Accelerated Life Testing; BMGF – Bill and Melinda Gates Foundation; DOE – Design Of Experiments; HALT – Highly Accelerated Life Testing; NMT – Nano Membrane Toilet; PD - Product Development; RTTC – Reinvent The Toilet Challenge; UCD - User Centred Design; UDDT – Urine Diversion Dry Toilet

## Introduction

To develop novel complex products, e.g. non-sewered sanitation technologies like Cranfield University’s Nano Membrane Toilet (NMT), fundamental research and creative design techniques have to be performed in combination, and building and testing physical prototypes is a crucial part of this process ( Tahera *et al.*, 2015). Larsen *et al.* (2016) acknowledge that, for technologies addressing urban water challenges, testing of technologies has to occur with a variety of methods to ensure robust, affordable, accepted and applicable solutions. Camburn *et al.* (2017) point out, that prototyping is most well-known for design refinement.

What constitutes a complex product can vary depending on the research question. A common definition is that a complex product “consists of a set of sub-products”, where “a sub-product may represent a specific piece of equipment, a business service, or a software-based service […]. Sub-products can also be composite, in the sense that they may consist of other sub-products” ( Afsarmanesh & Shafahi, 2013). According to Luo *et al.* (2016), developing complex products often has a multidisciplinary character, and research and development is complicated. Hobday (1998) emphasizes the numerous dimensions of complexity a product can have: “The term `complex' is used to reflect the number of customised components, the breadth of knowledge and skills required and the degree of new knowledge involved in production, as well as other critical product dimensions.”, and, building on this, Ljunggren Söderman & André (2019) add a “product chain dimension” and a “temporal dimension” to describe product complexity. For the purpose of this paper, a complex product is *a product that comprises multiple sub-products and technologies, thus requiring a greater development effort than simple, mass-produced products.* An example of such greater development efforts can be the use of a large number of physical prototypes.

Ulrich & Eppinger (2016) define a prototype very broadly as “an approximation of the product along one or more dimensions”. They furthermore identified four purposes of prototypes: learning, communication, integration, and milestones. Prototypes can also be categorised by how closely they resemble the final product, i.e. their fidelity ( Mccurdy *et al.*, 2006). Another important distinction of different categories of prototypes is between virtual and physical prototypes, and the use of virtual prototypes, or simulations, has increasingly gained importance in the past decades ( Tahera *et al.*, 2014). However, as the complicated interactions between the sub-products of a complex product will often be too difficult to simulate, this paper is focused on the testing of physical prototypes, as opposed to “the solution of analytical models and numerical approximations” ( Boës *et al.*, 2017).

In the context of prototype testing, the main purpose of a prototype is learning. Tronvoll *et al.* (2017) write about the use of prototypes: “A prototype experiment often targets generating knowledge about different attributes of a proposed design which is not identified by simple reflection.” Similarly, the IEEE (1990) gives a definition of testing, shared by ( Tahera *et al.*, 2019), as “an activity in which a system or component is executed under specific conditions, the results are observed or recorded, and an evaluation is made of some aspect of the system or component.” In combination, we define prototype testing as *knowledge-generating activity in which a prototype is executed under defined conditions, the results are observed or recorded, and an evaluation is made of some attributes of the prototype.* Prototype tests, like most activities in Product Development (PD) processes, can be seen as risk-reduction tasks ( Keizer & Halman, 2009; Unger & Eppinger, 2011).

There is ample literature advising on how to design and build prototypes according to testing needs (e.g. Camburn *et al.*, 2015; Menold *et al.*, 2017), and on testing strategies that aim to optimise the time and number of prototype tests (e.g. Al Kindi & Abbas, 2010; Qian *et al.*, 2010; Thomke & Bell, 2001). Camburn *et al.* (2017) thoroughly review the literature regarding strategies, techniques, and guidelines of prototyping. However, the question of *how* to test prototypes is seldom answered ( Tahera *et al.*, 2015), especially not with consideration of the entire PD process. Batliner *et al.* (2018), for instance, complain about the under-representation of general testing methodology in engineering literature impeding its integration into an engineering design curriculum. The planning of prototype testing, and communicating these plans, can therefore be difficult in the multi-disciplinary groups working on a PD project like for the NMT, and a tool to aid in these activities could be of great use. While different aspects of testing like iteration ( Wynn & Eckert, 2017), usability testing ( Unger Unruh & Canciglieri Junior, 2018), design of experiments (DOE) ( Ilzarbe *et al.*, 2008), Reliability Testing ( Bhamare *et al.*, 2007; Zhang *et al.*, 2014) and international standard compliance ( Shin *et al.*, 2015; Tyas, 2009) are well understood in their respective fields, a synthesis of these aspects could be used to develop a holistic test-planning tool for products like the NMT. Keller *et al.* (2006) discuss the difficulties faced by people developing large and complex products keeping an overview over the entire project and communicating their work to colleagues. They propose an improved way to visualise design processes to overcome this problem. Similarly, a visual tool that achieves synergy of different aspects of testing prototypes could be useful in planning and communicating testing efforts for the development of a complex technology like the NMT. The result could be more effective tests yielding more valid and useful data, as well as an increase in efficiency throughout the PD process. Future studies could use this tool developed from existing literature as basis to further develop it towards general applicability for prototype testing in PD by drawing from unreported expert knowledge of testing and engineering design.

This paper aims to review various aspects of testing prototypes to then propose a visual test planning tool to facilitate planning and communication of prototype testing for the development of the NMT.

## Methods

### Case study: The NMT, a complex product

Sanitation, the containment, transport, and treatment of human excrements, is a topic of high significance for human development ( Jahan, 2016): UNICEF (2017) stress the importance of safe sanitation for children’s health and that improving (access to) sanitation could reduce child mortality. Lack of sanitation has been linked to reduced cognitive development in children ( Sclar *et al.*, 2017) as well as stunting, caused by environmental enteric dysfunction ( Budge *et al.*, 2019), and to a risk of assault, particularly for women and girls practicing open defecation ( Jadhav *et al.*, 2016; Miiro *et al.*, 2018).

Urban sanitation poses particular difficulties, due to the lack of piped water and prohibitively high cost of sewer systems ( Cobbinah & Poku-Boansi, 2018; Parnell *et al.*, 2007). The most commonly promoted sanitation systems in cities of the Global South involve toilets that use little to no water, i.e. dry toilets, and store the faecal material onsite. Examples of this are pit latrines, pour-flush toilets, urine-diversion dry toilets (UDDT), and septic tanks ( Semiyaga *et al.*, 2015). When full, these toilets are emptied, and the faecal sludge is transported and either treated and reclaimed or discharged, or discharged without treatment. However, there are problems with these sewer-less sanitation services, such as high fees for emptying services, collection and transport trucks not being able to access the houses, high transport costs to treatment facilities or the altogether lack of such facilities ( Strande, 2014). Another obstacle for the success of these technologies is public acceptance: Users can consider the pedestals of dry toilets uncomfortable, dirty, or malodorous, and they may worry their children could fall into the pit ( Mkhize *et al.*, 2017; Roma *et al.*, 2013).

To find a solution to these problems, the Bill & Melinda Gates Foundation (BMGF) initiated the *Reinvent The Toilet Challenge* (RTTC) to “create a toilet that:

- Removes germs from human waste and recovers valuable resources such as energy, clean water, and nutrients,
- Operates “off the grid” without connections to water, sewer, or electrical lines,
- Costs less than US$0.05 per user per day,
- Promotes sustainable and financially profitable sanitation services and businesses that operate in poor, urban settings, [and]
- Is a truly aspirational next-generation product that everyone will want to use – in developed as well as developing nations.”

**Figure 1.**
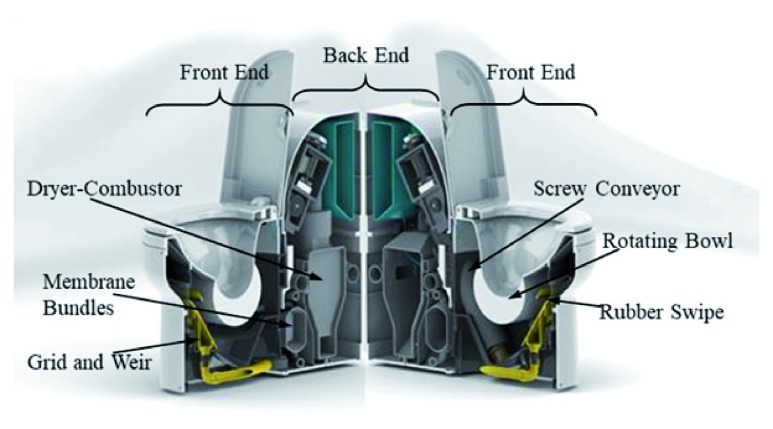
Conceptual schematic of the NMT and its components. The front end comprises the mechanical flush with its rotating bowl and rubber swipe, the collection tank with the grid and weir and the screw conveyor. The back end consists of the dryer, the combustor, and the membrane bundles.

**Figure 2.**
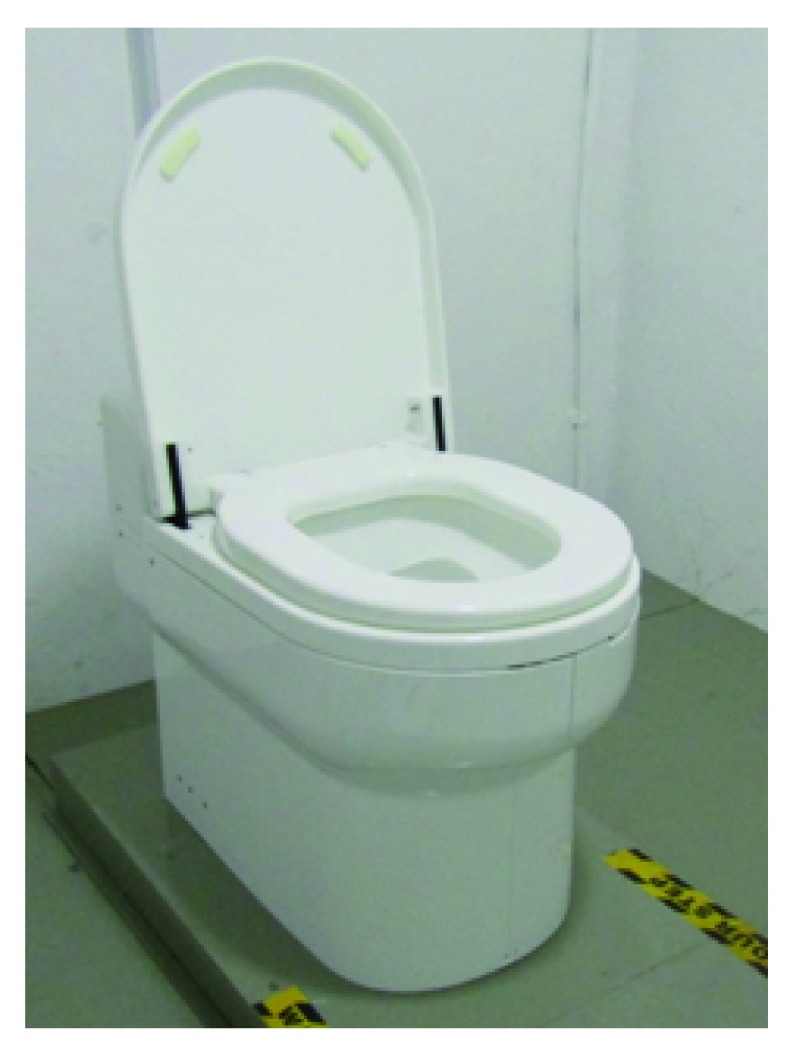
Nano Membrane Toilet front-end prototype.

As result, research institutions and companies worldwide are now developing waterless, non-sewered sanitation technologies ( Bill & Melinda Gates Foundation, 2013). The reinvention of the toilet requires unconventional thinking. Niemeier *et al.* (2014) discuss the challenges of building technologies for the development context: “If we are to resolve global inequities in access to innovations that improve health, we must adopt new approaches to engineering design that reflect the unique needs and constraints of low-resource settings”. They further mention how “efforts like the [RTTC] reflect the kind of integrative thinking that must occur at the beginning of a design initiative […]”. One example of a reinvented toilet is the NMT, conceived by researchers at Cranfield University ( Parker, 2014). The NMT is a household-level, onsite sanitation system that looks similar to a porcelain water flush toilet ( Figure 1 and Figure 2). It uses combustion and membrane processes to treat the mechanically separated solid and liquid waste streams. With all its components, the NMT would not just replace the currently existing dry toilet technologies, but also the associated faecal sludge management services, thus offering a form of safely managed sanitation ( WHO & UNICEF, 2017). It is not simply a human waste receptor, but rather a miniature faecal sludge treatment facility.

Naturally, there are numerous considerations to be made during the development of such a technology. The NMT combines entirely novel technologies with already existing ones. However, even for the well-established technologies, their application for this specific purpose is novel, and requires further research in order to miniaturise, integrate and optimise for off-grid functionality. At the same time, from a user’s perspective, not much should change when transitioning from using another dry toilet or a porcelain flush toilet to the NMT. To fulfil the RTTC’s demand for an aspirational design ( Bill & Melinda Gates Foundation, 2018), it should be comfortable, appealing, and simple to use. It should take into account the preferences and customs of users from a diverse range of cultural backgrounds. Fejerskov (2017) emphasizes the importance of considering the users of a newly developed technology: “[…] a technology developed in isolation from those who are supposed to benefit from it cannot be expected to wield predictable outcomes.” There is research on the preferences of toilet users in various contexts, from industrial nations like the Republic of Korea ( Lee, 2019) and Canada ( Morales *et al.*, 2017), to a focus on elderly users ( Dekker *et al.*, 2011) to low- and middle income countries ( Austin-Breneman & Yang, 2017; Nelson *et al.*, 2014). Hence, developing the NMT entails developing a user-friendly user interface—in software development projects this would be called “front end” ( Reza & Grant, 2007)—and a “back end” comprising several sub-technologies, and integrating them into the overall system.

The RTTC has thus led to an unusual case of PD at this scale: It asks for a product that connects existing notions of a toilet’s function and design with never-before-seen technologies. A viable solution to the problems associated with dry sanitation must simultaneously satisfy users’ ideas of aesthetics and comfort, and adhere to high standards of safety and reliability.

The NMT is a complex product, which is, in fact, a combination of sub-products that have to be developed individually and then integrated into the overall system. The original design brief for the NMT was the RTTC, which included important user-centred objectives of aspirational design and affordability, as well as objectives aiming at sustainability and at solving the problems of urban non-sewered sanitation ( Bill & Melinda Gates Foundation, 2013). From this, initial design ideas were conceived involving membrane treatment of liquids and water recovery through condensing beads, as well as the drying and coating of solids ( Parker, 2014). Later design stages discarded the condensing beads and a combustion process was devised to replace the coating of solids. Considering that the user of the toilet would usually not interact directly with the treatment processes, these were not subjected to user testing yet. The pedestal, the part of the toilet with which the user interacts, mainly differs from a porcelain water flush toilet in its mechanical flush. It was developed as the result of studies among potential users in Ghana and subsequent agile innovation processes ( Tierney, 2017). Several iterations of prototypes were produced to develop a mechanical flush, until it could be tested in real-use scenarios.

This mechanical flush – a rotating bowl and rubber swipe activated by moving the toilet lid – separates the user from a tank underneath the toilet pan ( Tierney, 2014; Tierney, 2017). Solids are separated through settling and displacement, transported by an screw conveyor ( Mercer *et al.*, 2016) and subsequently dried and combusted ( Fidalgo *et al.*, 2019; Onabanjo *et al.*, 2016), while the liquid fraction is extracted through a weir and purified through membrane processes ( Kamranvand *et al.*, 2018; Wang *et al.*, 2017), driven by the heat of the combustion, which is transferred via a heat exchanger ( Hanak *et al.*, 2016). The toilet pedestal, including the mechanical flush, the screw conveyor, and the liquid weir are considered the NMT’s front end. The dryer, combustor, and membrane components are considered its back end. The NMT is envisioned to be independent of water- or sewer connections and energy neutral, or even have a positive net power output ( Kolios *et al.*, 2018). However, the back end components have not yet been integrated and combined with the front end to produce a fully functioning prototype of the NMT. Such tasks are envisioned to be conducted in the near future.

At the moment, all sub-products which the NMT comprises are in an iterative phase of building and testing prototypes. The front end has been re-designed as result of field tests involving target users of the NMT ( Hennigs *et al.*, 2019). Prototypes of the dryer and combustor ( Jurado *et al.*, 2018) have been tested in the lab in several iterations. In addition, the recovery of electrical energy by reverse electro-dialysis is under investigation.

This means that the individual components and sub-products of the NMT are developed enough to plan for integration of all sub-products into a complete prototype. Such a prototype would then be tested in laboratory tests, and once its safe operation was sufficiently proven, it could be deployed for user-centred field tests. The aim of such tests, and concurrent further sub-product improvements, would be to optimise the operational settings of the entire system. The recently published ISO 30500 standard ( ISO, 2018) could provide the benchmark performance values the prototype has to achieve. Once the prototype meets these values, its design can be polished for usability and manufacture. Final reliability and user tests of this polished design would ensure the NMT’s usability and reliable functionality throughout its lifetime, and when passed, allow this design to confidently be tested for ISO 30500 standard-compliance, making it a market-ready product.

Consequently, there are still numerous prototype tests which need to be planned and conducted. The development and testing of prototypes of the NMT’s various sub-products to date have not been guided by a visual test planning tool. Instead, prototypes of components were developed and tested by the teams working on these components. In the case of the front end, a prototype was developed by one team and then tested by another ( Hennigs *et al.*, 2019). It could therefore be possible that the prototype tests to date could have been conducted more effectively had they been planned in a more coordinated manner. Similarly, the communication within and between the various teams working on the NMT could benefit from a more consolidated terminology and shared understanding of the development process and the associated testing activities. A visual test planning tool could thus facilitate and improve the planning and communication of testing activities in the future.

### Literature review

Using our own publications and those of our colleagues on the project, the development history of the NMT was established as that of a complex product and outlined in the section above. Subsequently, the Scopus and Google Scholar databases were used for an exploratory search of peer reviewed literature, with a focus on literature reviews covering a range of publications, to advance the understanding of the broad field of prototype testing in PD. Search terms included *review, prototyping, prototype testing, product development, technology development, testing,* and others. Using these terms in various combinations, promising publications were identified and studied individually. In the search, several repeatedly occurring aspects of testing prototypes were identified and then further investigated in a more targeted search on the same databases. The aspects were *types of prototype tests, phases of the PD process, iteration, usability testing and user centred design, reliability testing, testing for standard compliance, design of experiments, back end and front end testing, and visualisations of PD processes*. They were chosen for further investigation because of their repeated occurrence in the literature and their apparent applicability for the context of this paper. Again, the focus of the resulting search was on reviews of the existing literature rather than original work, as the aim was to obtain a wide understanding of multiple fields of study, rather than an in-depth analysis of a single one. Similar search terms were used, with the addition of the identified testing aspects. The identified literature was then analysed to extract the information relevant to prototype testing activities, particularly for the development of complex technologies. This analysis yielded the section ‘relevant aspects of prototype testing’.

### Creating a visual test planning tool

A visualisation of the prototype testing processes for the PD of the NMT was then conceived, with the aim to consolidate the collected information. A simple linear PD process model was chosen, divided into three phases, with parallel strands of testing for the front end and back end, considering different types of testing, DOE, usability testing, reliability/durability testing, and international standards at different stages of the process, and possible iteration loops throughout the process.

## Relevant aspects of prototype testing

While the route to technological maturity of a product may seem straight-forward, each step can involve extensive preparations and cooperation among multiple teams. It is sensible to separate the PD process into distinct phases, which involve distinct activities ( Rubin & Chisnell, 2008). To maintain an overview of the progress, a visualisation of the entire PD process can be valuable ( Keller *et al.*, 2006). “Iteration is a fact of life in any [PD] project”, particularly for complex products ( Wynn & Eckert, 2017). It should thus be considered when planning testing activities, as well as the fact that these tests can be of different types, in different settings, with different aims and methodologies ( Boës *et al.,* 2017). In the example of the NMT, prototype integration not only requires a sufficient level of maturity of all sub-products, but also operational process control to connect all sub-products with each other considering their complex interactions. This requires extensive knowledge of all sub-products’ operational conditions, which may be acquired in tests that Boës *et al.* (2017) would classify as experiment tests, using statistical DOE ( Ilzarbe *et al.*, 2008). Reliability and durability estimation methods are needed to ensure the system’s reliability and durability throughout its lifecycle ( Bhamare *et al.*, 2007), and often national or international standards exist to ensure the technology is safe to use ( Feo-Arenis *et al.*, 2016). Additionally, as mentioned in the introduction, prototype and system tests need to be centred on the target users of the technology. If they do not want to use a novel toilet, it will fail to have a positive impact on the sanitation crisis. Methods of UCD, e.g. usability testing, can be used to avoid such failures ( Unger Unruh & Canciglieri Junior, 2018). In complex products, it is likely that the users will only interact with parts of the product, the user interface. It can therefore be sensible to consider testing efforts separately, as is common in software development, where front end and back end are tested separately ( Bertolino, 2007).

In the following subsections, the principles of these aspects of testing prototypes are presented.

### Types of prototype tests

Boës *et al.* (2017) define testing in the context of PD as “exposing a physical system to a condition or situation in order to observe the system’s response.” They then clarify the physical system as a representation of the product or one of its components, the condition or situation as a “use case as a whole or its effect on a subsystem”, and the system’s response as “the performance of a desired function [or] an undesired failure mode.” Using this definition, they propose four categories of testing activities according to the type of knowledge that is generated. First, trial and error tests can be used to gain a basic understanding of the development project and to explore the design space. Secondly, experiment tests resemble experimental work in fundamental scientific research in their structured approach in order to identify influencing factors and develop “necessary system knowledge”. Thirdly, verification tests are usually pass/fail tests to determine if the system- or component prototype fulfils the requirements set at the beginning of the PD process. Lastly, validation tests determine whether the product addresses the underlying user needs, rather than the requirements set by the product developer. They are commonly conducted with a fully functional prototype.

Camburn *et al.* (2017) comprehensively review the state of the art in prototyping techniques, strategies, and guidelines, and devise a visual framework to connect prototyping objectives to techniques. They clearly distinguish prototypes from design concepts by stating that prototypes are always tied to tests. They discuss literature on preparing for prototyping, enhancing prototype performance, reducing cost and time, and fabricating prototypes, before reflecting on prototyping science. While they don’t specifically identify types of prototype tests, they do list frequently cited prototyping objectives, namely active learning, exploration, communication, and refinement. They further develop scales of prototype distinction, i.e. “ *system (isolated or integrated); media (virtual or physical); requirements (relaxed or final); and scale (reduced or final)”*. They also discuss iterative prototyping, parallel prototyping, requirement relaxation, subsystem isolation, scaled prototyping, and virtual prototyping as individual prototyping techniques.

There seems to be the implication that there are different types of tests being conducted throughout the PD process that differ in their level of formality, in their approach, and in the knowledge they are designed to produce. A similar observation can be made about the prototyping tests for the NMT. We have identified three distinct types of prototype tests conducted so far, namely user tests, laboratory tests, and field tests. These types of tests can coincide and overlap: just for one generation of a front-end prototype, Hennigs *et al.* (2019) conducted user surveys and interviews (user tests), photography and image analysis (laboratory tests), and field tests on the material choice of a rubber swipe and the long-term robustness of the prototype (field tests). A fourth type of tests are international standard tests. In the case of the NMT, these would be described in the standard *ISO 30500:2018 Non-sewered sanitation systems* ( ISO, 2018).

### Phases of the product development process

In the case of usability testing, Rubin & Chisnell (2008) distinguish three phases of testing: First, exploratory tests are conducted in the early stages of PD, to test its basic design, i.e. whether users find it intuitively appealing. Then, assessment tests, conducted about halfway through the development process, expand the knowledge on the product’s usability, i.e. whether users can perform the intended tasks on the product. Lastly, the validation/verification tests at the end of the cycle tend not to inform further iteration, but rather confirm that all previously identified problems have been resolved, and that the entire product can be used as intended. In these phases, the basic design, early to well-developed prototypes and the final product are tested on potential users to identify their likes and problems.

In his Stage-Gate model, Cooper (1990) considers five stages between a product idea and a “post implementation review”, namely *1. Preliminary assessment; 2. Detailed investigation (business case) preparation; 3. Development; 4. Testing and Validation; and 5. Full production and market launch*. Royce’s (1970) Waterfall model comprises seven steps to develop a large computer program: *1. System requirements; 2. Software requirements; 3. Analysis; 4. Program design;5. Coding; 6. Testing; and 7. Operations.* Similarly, other models of PD processes are also sorted into phases or steps ( Boehm, 1988; Forsberg *et al.*, 2005). While testing is a single distinct step in most of such models, the various types of prototyping activities and prototype tests throughout the PD process could also be imagined to be separated into phases as the product’s maturity increases. Rubin & Chisnell’s (2008) three phases (exploration, assessment, validation/verification) provide a sufficient level of distinction for the present work on the NMT.

For this purpose, the objective of the exploration phase is to explore potential solutions to the design brief, to discard unviable ones, and to gather an understanding of the required development process, i.e. to develop the questions that need to be answered in the assessment phase.

The objective of the assessment phase is to expand the knowledge about the potential product solutions, to answer the questions developed in the exploration phase. The outcome of this phase should be one single, functional prototype.

The objective of the verification and validation phase is to ensure that all questions have been answered, that the product functions as expected and is, in fact, a solution to the design brief.

### Iteration

Iteration occurs throughout the PD process and can have different causes and outcomes ( Wynn & Eckert, 2017). With its earliest forms dating back to the 1930s ( Larman & Basili, 2003), the *sequential testing and refinement of a prototype* ( Christie *et al.*, 2012) can be welcomed as a driver of positive design change, or seen as a wasteful, costly delay in a PD project ( Ballard, 2000; Le *et al.*, 2010; Wynn & Eckert, 2017), but it is undeniable that iteration occurs in nearly every PD process, particularly for complex products ( Wynn & Eckert, 2017).

It is common to develop software user interfaces iteratively ( Nielsen, 1993). For complex physical products, every iteration-step of building and testing a prototype can be associated with high costs ( Tahera *et al.*, 2019). It is therefore important to consider when and how many iteration-steps should be undertaken. Camburn *et al.* (2017) emphasize that iteration should occur often and early in the PD process, and should be encouraged the higher the potential for performance increase and the lower the cost per iteration. The higher the product’s maturity, the higher the cost of iteration will likely be. There is a multitude of publications discussing the complex nuances of iteration in PD, and Wynn & Eckert (2017) offer a comprehensive overview and terminology of this field of research: They differentiate between *micro-level* and *macro-level* iterations, as well as between three iterative functions of *progress* toward completion, *correction* of errors, and *coordination* of actors, decisions, or workflows. They assign multiple *iterative stereotypes* to each function: Progressive iteration comprises the stereotypes *exploration, concretisation, convergence, refinement, and incremental completion.* Corrective iteration covers *new work, rework,* and *churn.* Lastly, coordinative iteration encompasses the stereotypes *governance, negotiation, parallelisation, comparison,* and *concentration* ( Wynn & Eckert, 2017). This illustrates the complex role that iteration plays in PD.

For the development of an NMT testing tool it is mainly important to know that, while iterative loops can occur throughout the PD process, they value for cost reduces as product maturity increases ( Wynn & Eckert, 2017), and to consider which test results should trigger or prevent an iterative loop. With the main goal of the PD process being a marketable product, any proof that a prototype does not represent such a marketable product should be a trigger of iteration. Failed tests, like the shortfall against benchmark values, can be seen as such a proof. International or internal standards can provide such benchmark values. Cost is a deciding factor, and will have to be considered by a project manager when deciding whether to iterate further or not. However, the complex calculations weighing iteration-cost against the cost of having a less well-tested product would go beyond the scope of the present work.

### DOE

Laboratory-based tests commonly involve the observation of a (sub-) product’s condition and/or outputs in relation to its inputs. As mentioned above, a comprehensive understanding of the systems’ outputs respective to their inputs and process variables is required. Often, there are several inputs and/or outputs for one component, and inputs can interact with each other to create second- or higher-order effects on the outputs ( Montgomery, 2009). For example, potential factors that can affect the processes in a combustion chamber are the amount of fuel, its flux, its moisture content and calorific value, the process temperature as well as the flux, pressure, temperature, humidity, and oxygen content of the inflowing air ( Jurado *et al.*, 2018).

To test all inputs and their interactions, across their entire range, is either very difficult or impossible. It would take hundreds of tests to assess every factor’s influence on the combustion process. Some factors cannot be controlled; some cannot be changed without affecting another.

Based on the work of statistician R.A. Fisher ( Fisher Box, 1980; Yates, 1964), DOE uses statistical approaches to address such problems, to minimise the time and effort required for a set of experiments while maximising the validity, reliability, and replicability of information gathered from them. The basic principles of DOE, initially developed for agricultural research, are ( Cortes *et al.*, 2018; Fisher, 1935):

- **Factorisation:** the variation of several experimental factors at once in order to reduce the number of experiments to run.
- **Replication:** the repetition of an experiment with the same settings for experimental factors (treatments) in order to estimate the experimental error.
- **Randomisation:** the random application of treatments and order in which experiments are run, to validate the assumption that the observations and errors are independently distributed variables.
- **Local control of error, or blocking:** the subdivision of experimental runs into homogenous blocks in the attempt to lessen the impact of errors introduced by controllable nuisance factors, e.g. male and female patients in medical drug trials.

It may occur that these principles have to be compromised to some extent for practical reasons, or that complex processes are to be investigated. Within the DOE-toolbox are methods such as split-plot design ( Kulahci & Tyssedal, 2017; Lee Ho *et al.*, 2016), fractional factorial design, response surface methodology, and random effects models ( Montgomery, 2009) for such cases. It is thusly possible to achieve a high level of understanding from comparably few experimental runs. For example, Ilzarbe *et al.* (2008) found in their bibliographical review of 77 DOE applications in the field of engineering, that with an average of 5.06 factors to be investigated, 77% of the studies achieved this goal with 30 or fewer experiments, and 50% with 20 or fewer.

DOE finds application in PD efforts of various kinds: Pineau *et al.* (2019) used a fractional factorial design to assess which design factors of coffee vending machines impacted the sensory experience of the product the most. Gumma & Durgam (2019) improved the structural performance of a car’s body using a multi-model DOE sensitivity study, including simulations and experimental model testing. Sano *et al.* (2019) studied Bayesian optimisation techniques to reduce the number of experiments necessary to obtain the information with which they could improve production parameters for orally disintegrating tablets.

Thus, DOE encompasses a wide range of statistical tools for planning how to conduct tests, and how to analyse the results later on, to maximise the statistical validity of the lessons learnt. It does, however, not give any advice on what to test, or why. Other problems with DOE can be that statistical models developed through its use do not accurately reflect the observed processes ( Deaconu & Coleman, 2002), or that it gives false credibility to results that stem from badly conducted experiments or the incorrect application of DOE principles. For example, modern technical processes and systems can and must be tested differently to fields of crops ( Collins *et al.*, 2011).

### Reliability and Durability Testing

Reliability estimation, a part of reliability engineering, comprises reliability tests and the analysis of the data gathered in those tests. Kapur & Pecht (2014) define reliability as “the ability of a product to function properly within specified performance limits for a specified period of time, under the life-cycle application conditions”, i.e., how long a product functions without needing repair. This means that reliability tests are carried out to assess the likelihood of the product—or its components—failing over time. They are usually conducted on prototypes of high maturity, on randomly selected products fresh off the assembly line, or even products that have been in use for a certain time. Similar to DOE, statistical approaches are used to calculate a level of confidence with which a failure will occur in a given time ( Kapur & Pecht, 2014).

Durability is a measure of a product’s lifetime, i.e. the how long the product can be used until repairs become more expensive than replacing it ( Garvin, 1996). In other words, “durability is a particular aspect of reliability” ( Tahera *et al.*, 2019). This implies that, while the calculation of durability will involve repair costs and other economic factors, the durability tests to assess likelihood of failure will often be similar or identical to reliability tests. This similarity is reflected in existing literature, where both terms are often used in conjunction ( Lee *et al.*, 2015; Munson *et al.*, 2018), and sometimes seemingly synonymously ( Bluvband, 2012; Jimenez, 2017).

For both, the challenges lie in accelerating the product’s lifetime: It is not feasible to test a statistically significant number of product units over a number of years in order to assess their reliability and durability for this timespan. Therefore, a reliability/durability engineer attempts the realistic emulation of real use scenarios and environmental conditions in a shortened period of time by applying potential stresses, like shock, vibration, or climatic conditions in rapid succession, periodically, or simultaneously ( Cheon *et al.*, 2015; Donovan & Murphy, 2005; Zanoff & Ekwaro-Osire, 2010). For this aim, accelerated life testing (ALT) is used to determine a product’s time until failure by compressing its lifetime in a short period, usually weeks or months. Highly Accelerated Life Testing (HALT), in contrast, is a technique to determine the most likely failure points of a product but compressing its lifetime into a very short period, usually hours or days ( Silverman, 2006).

Difficulties with these approaches lie in the complexity of combined stresses and failure modes, particularly on complex physical products. It is difficult or impossible to “model multiple (or competing) failure mechanism[s] to support reliability testing methods” ( Bhamare *et al.*, 2007). Furthermore, reliability / durability engineering does not consider the user’s experience, but rather focuses solely on the product’s reliable functionality. Thus, reliability / durability tests may miss important inputs, as (mis-)use is an important factor in the lifetime of a product, and important outputs, as the user experience may be a more important factor in design changes than increased reliability and durability. For example, a sturdier handheld device may be more reliable and durable, but too heavy or impractical to use.

### Testing for technical standard compliance

International “technical standards are established norms or requirements applied to technical systems. They are a crucial aspect of almost all industries […]” ( Shin *et al.*, 2015). They play an important role in technology development ( Østebø *et al.*, 2018), by providing “a benchmark for quality and acceptability in the market place” and guidance on the “safety, reliability, efficiency and interchangeability” of products ( Tyas, 2009). The testing procedures and performance requirements outlined in technology standards form the basis for the process of ensuring a product is compliant with the standard before being released to market. This does, however, not mean that the first time the standard should be consulted is at the end of the PD process. Instead, the performance, safety and other requirements provide the benchmark to which even early prototypes can be compared, and the testing procedures and-protocols can be adapted or used directly to test prototypes of sufficient technological maturity.

Examples of standards being used as benchmarks during product testing are for a wireless fire alarm ( Feo-Arenis *et al.*, 2016), packaging of products ( Nolan, 2004), or sensor interface circuits for the automotive industry ( Ohletz & Schulze, 2009). Another example is the ‘syngina test’, the standardised test for tampon absorbency. The standard was developed by the American Society for Testing and Materials, when a link between tampon size and toxic shock syndrome was discovered, but customers could not reliably buy tampons of similar absorbency from different brands ( Vostral, 2017).

While international standards provide this much-needed guidance, it is important to remember that they are not infallible and may overlook important aspects especially of innovative technologies. For example, Mjör (2002) noted that, for dentistry equipment, “parameters measured in the standards are often not predictors of clinical performance”, and often lacked clinical backing. For the case of the ‘syngina test’, Vostral (2017) discusses the issue that the test is merely a very coarse approximation of a menstruating human body. Narayanan & Chen (2012) discuss the fact that in the early stages of competing, similar technologies, the setting of a standard can result in a “winner-take-all outcome”, as seen in the competition between Betamax and VHS video systems. Moreover, Hu (2010) lists potential threats that international standards could pose to innovation, such as exclusion of innovative start-ups from the market and lack of incentive for leading companies to innovate beyond a minimum-standard level of quality, but also mentions that the benefits of standards toward innovation outweigh the limitations.

### The ISO 30500 Standard

The attempt to assist innovation through standards can be applied to the development of sanitation technologies as well: the ‘International Organization for Standardization’ (ISO) recently published the standard *ISO 30500:2018 Non-sewered sanitation systems*. It “specifies general safety and performance requirements for design and testing as well as sustainability considerations for non-sewered sanitation systems”, and thus aims “to support the development of stand-alone sanitation systems […] and promote economic, social, and environmental sustainability […] ” ( ISO, 2018). In the document, requirements for performance, materials, safety, maintenance, and sustainability are listed and testing procedures are described in great detail. The ‘Annex A – Test methods and additional testing requirements’ comprises 33 pages, and the main document 34. The range of tests covers a comprehensive list of aspects concerning the safety, quality, and usability of non-sewered sanitation systems, but does not necessarily account for the statistical variation in measurements, as would be considered in DOE. For example, only one unit of a new sanitation system is to be tested for the ISO 30500 standard. Similarly, the standard does not fully support a UCD approach. While the ease and safety of use are described as requirements, and consideration is given for variations in cultural requirements like the distinction between users preferring the squatting or seating positions, the standard cannot account for the broad variety of user preferences according to their physical, cultural, and social needs. Such considerations are given in UCD.

### Usability testing and User Centred Design

The term ‘User Centred Design’ (UCD), first coined and publicised by Norman & Draper (1986) in the context of software design, encompasses a collection of processes and methodologies that follow the basic principles of a human-centred approach, which are now described in the international standard ISO 9241-210:2010 ( ISO, 2010):

- *The design is based upon an explicit understanding of users, tasks and environments.*
  ➔ Identify all relevant stakeholders, their needs, and the context of use, i.e. the characteristics of users, tasks, and environment.
- *Users are involved throughout design and development.*
  ➔ Users are an important source of information about context of use. The participants should reflect the target users’ (range of) characteristics. The type and magnitude of participation will likely change throughout the development process.
- *The design is driven and refined by user-centred evaluation.*
  ➔ Gather user feedback on designs, e.g. prototypes, to detect unknown challenges or requirements. The final product can similarly be tested to ensure the UCD was a success. Long-term issues can be uncovered through user feedback after market-release.
- *The process is iterative.*
  ➔ As described above, repeating certain steps of the design process while building on the learnings of the previous repetition is widely accepted as a successful method of progressively improving the design ( Wynn & Eckert, 2017).
- *The design addresses the whole user experience.*
  ➔ The user experience is influenced by the technology’s functionality, performance, and user interface, as well as the user’s individual characteristics, skills, and previous knowledge. To improve it, all these factors need to be considered, and the user-technology interaction should be adjusted accordingly.
- *The design team includes multidisciplinary skills and perspectives.*
  ➔ While the interdisciplinary, and often international, nature of teams collaborating on PD projects can be the cause of conflict initially ( Yim *et al.*, 2014), it is widely accepted that the combined knowledge and skillset of multidisciplinary teams are beneficial to their success ( Edmondson & Nembhard, 2009).

Usability testing, is only one, albeit essential, part of the entire UCD process ( Bastien, 2010), and comprises in itself a range of possible tests: from complex experimental designs as produced with DOE methods and involving large numbers of participants to rather informal tests with a single potential user as participant. Considering the aim of such testing is often to obtain qualitative information for the design process, rather than obtaining statistically relevant design parameter values, less formalised, qualitative methods are the focus of Rubin & Chisnell’s (2008) ‘Handbook of Usability Testing’. While there are cases of user tests designed using DOE principles (e.g. Jensen *et al.*, 2018), these remain the exception, as the more common approach for user tests seems to be a qualitative one ( Bastien, 2010) .

Usability testing and UCD appear to be particularly important for products that have a high degree of complexity but are used by a broad spectrum of users, with varying degrees of expertise, for example a tubeless insulin pump ( Pillalamarri *et al.*, 2018). Another application would be for products specialised for a certain user group, like a motorcycle tool for one-handed users ( Sudin, 2013). However, a large portion of usability tests is still conducted in software development, for example for a drill rig control system ( Koli *et al.*, 2014).

### Distinction between front end and back end – lessons from software development

For the case of the NMT, a distinction between the toilet seat, bowl, and flush, i.e. the “user interface” or front end, and the treatment system, or back end, can be made. This is analogous to software products like web sites ( Chen & Iyengar, 2003). The front end and back end of a system require different testing in the PD process ( Bertolino, 2007; Sánchez Guinea *et al.*, 2016; Sneed, 2004). For example, while the front end is the part of the product with which the users will interact, the back end is usually only of indirect concern for them, as long as everything operates as expected. Hence, the front end should undergo user testing early on ( Chuang *et al.*, 2011), while the back end might only require limited user testing at later stages, to ensure maintainability by trained personnel. On the other hand, the back end of the NMT will likely require more extensive laboratory testing than the front end, as the treatment processes are more compicated than the user interface.

It might therefore be sensible to consider testing for the front end and back end independently, and in a separate step actualise integration testing of the individual components.

### Visualisations of PD processes

As mentioned above, visualisations can be beneficial for product developers to plan and communicate their work ( Keller *et al.*, 2006). Wynn & Clarkson (2018) give a very comprehensive overview of a large body of work on process models in PD, including visualisations thereof. They categorise models focusing “on the large-scale organisation and management of design and development” as macro-level models. Examples of these are well-known models such as the Stage-Gate model ( Cooper, 1990), the V-model ( Forsberg *et al.*, 2005), the Waterfall model ( Royce, 1970), and the Spiral model ( Boehm, 1988). While all these models differ in their philosophy, reflected in the shape of their visualisations, they have some common characteristics: They all describe a continuously progressing process towards the final product, and while they all mention testing at some point in this process, they do not seem to consider testing throughout its entirety. Generally, the importance of the role that testing plays in the various models reviewed by Wynn & Clarkson (2018) is not reflected in their visualisations. This further emphasizes the importance of developing a visualisation of testing activities in PD, not just for the NMT.

As the NMT project does not follow a specific PD process model, a visual description of the testing activities could conceivably be shaped in a number of ways. However, for the sake of simplicity, it seems sensible to base it on the progression of the project to date. Recently, two teams worked on designing and testing the NMT’s front end, and two teams were allocated to researching, developing, and testing the back end membrane and combustion processes respectively. As described in the previous section, a separation into parallel testing activities for front end and back end seems sensible. Furthermore, the entire process has progressed steadily, and even though iteration occurred, a linear model describes the overall development most aptly.

The end point of many models is the launch, or deployment, of the product, although some consider also the operation and maintenance of the product ( Wynn & Clarkson, 2018). For the case of the NMT, the envisioned final test of a prototype, one which is practically identical to the final product, is envisioned to be the testing for compliance with the ISO 30500 standard, conducted by a licensed laboratory. Therefore, this test would signify the endpoint of a visualisation of the NMT testing strategy.

### Summary

A testing tool for the development of a novel sanitation system should consider all aspects to provide a comprehensive overview of the considerations that need to be made when planning prototype tests. We believe that a combination of existing concepts – not only in testing – is a common occurrence in PD. An example is Lean Six Sigma, a now established concept itself, which combines the two management methodologies ‘Lean’ – a methodology to “remove non-value activities from the [PD] process” and ‘Six Sigma’ – a methodology to reduce variability and thus defects and errors in the process of concern ( Alexander *et al.*, 2019). Furthermore, since the dawn of computer aided engineering, a combination of virtual and physical testing has been ( Van Der Auweraer & Leuridan, 2005) and continues to be promoted by experts ( Tahera *et al.*, 2014). Likewise, the testing practice in private enterprises is likely to be more experience-based and will often combine various concepts to varying degrees. However, a visualised combination of different aspects appears to be a novel conclusion.

To incorporate all discussed aspects, a tool for testing of prototypes in PD should thus:

- Have a visualised form,
- Consider different types of tests, for example user tests, laboratory tests, field tests, and international standard tests,
- Consider different phases of the PD process (e.g. *exploration phase, assessment phase, and validation/verification phase*), and how the knowledge sought by prototype tests changes in each phase,
- Consider iteration as a possibility throughout the PD process, while indicating that the decision to iterate is based on many factors,
- Consider front end and back end testing as distinct activities,
- Consider usability testing, and follow UCD principles, particularly in the development of the front end,
- Consider the use of standard compliance tests and standard requirements as benchmark for performance and safety,
- Consider the use of ALT and HALT methodology to identify weak points and estimate the product’s reliability and durability, and
- Consider the use of DOE-principles where possible to ensure statistical validity and efficient use of time and resources.

### Synthesis: A Visual test planning tool

**Figure 3.**
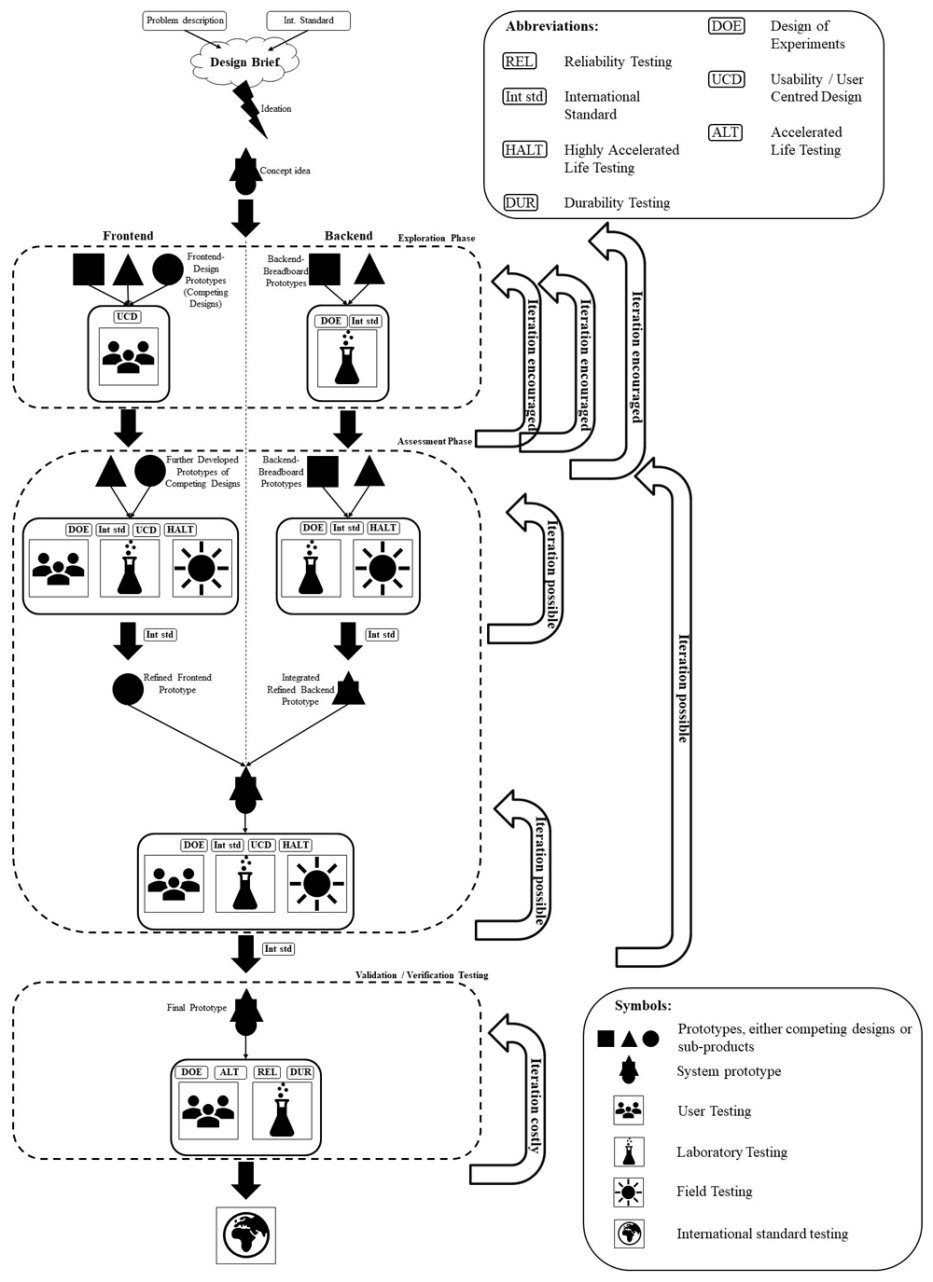
Visual test planning tool. A simple linear PD process model was chosen, divided into the three phases exploration, assessment, and validation/verification, with parallel strands of testing for the front end and back end, considering different types of testing, DOE, usability testing, reliability/durability testing, and international standards at different stages of the process, and possible iteration loops throughout the process. As value over cost per iteration decreases with product maturity, iteration loops are marked as *encouraged, possible,* and *costly* over the course of the three phases.

Derived from the considerations described above, a visual test planning tool for the NMT could look like the flow chart shown in Figure 3.

Starting from the problem description, and potentially using input from already existing international standards, a design brief is the first step of the PD process. This will prompt first concept ideas, thus beginning the exploration phase. Designers will then develop potential solutions to the problem, which will be realised in the first prototypes, both for the front end and back end. The front-end prototypes will often have low functionality and are mainly used to communicate the designers’ vision. They can then be tested with potential users, to assess whether the designers are ‘on the right track’, and their proposed solutions could be accepted by users. The back-end prototypes will likely be first breadboard prototypes of single components, to prove the viability of the concept ideas. Several competing designs might be tested simultaneously, and iteratively, to refine the initial designs. In this phase, more iterations should be encouraged, as producing and testing prototypes is relatively cheap and yields a lot of useful information.

In the following assessment phase, further developed prototypes can be constructed. These might be prototypes of sub-products or the whole product, and they are likely to already have a certain degree of functionality. It is on these prototypes, and increasingly developed iterations thereof, that a variety of tests will be conducted to learn about the technical, functional, and aesthetic aspects of the NMT. Using DOE and UCD methods, and tests from international standards as benchmarks, components and (sub)systems are tested in laboratories and field tests towards functionality and usability. HALT methods can be used to identify and mitigate likely failure points. While first tests will still be conducted on separate prototypes for the front and back end, user tests and functionality tests can be conducted simultaneously at later stages of sub-product maturity, when an integrated prototype is constructed. Several iterations are possible, until satisfactory component and system maturity is reached, and competing designs can be developed and tested simultaneously. It can be a difficult decision to define a cut-off point for further iterations. The developer has to have confidence that the entire system will safely function as intended. The minimum performance values of an international standard can provide helpful guidance to ensure this confidence.

In the final phase of validation and verification, a finalised design, maybe already produced on the product’s assembly line, is tested for reliability and durability using ALT methods. DOE methods can be applied to improve statistical validity of tests. Final user tests ensure all user-related problems have been mitigated. If no serious problems arise, the product can be sent to be tested towards compliance with an international standard. In the case of the NMT, this would be the standard *ISO 30500:2018 Non-sewered sanitation systems*.

There is a possibility that tests reveal problems which necessitate a return to much earlier stages of the PD process. However, this should be avoided by completing an appropriate number of test iterations during the exploration and assessment phases, as it would entail significant cost to iterate at such a late stage.

## Discussion

The visual test planning tool presented in Figure 3 gives an overview of the considerations to be made for planning and communicating testing efforts during the development process of the NMT. Tahera *et al.* (2015) stress the importance of testing in the PD process, and there is abundant research on the ideal number and timing of tests within PD (for example Al Kindi & Abbas, 2010; Thomke & Bell, 2001). However, there is a lack of publications focusing on how to plan and communicate testing throughout the PD process, which is attempted here.

The flow chart aims to combine several aspects of testing prototypes that, in their own field, are well-developed concepts with numerous publications and ongoing research on refining and advancing methods. It would be beyond the scope of this paper to attempt to outline more detailed descriptions of all aspects, and we therefore refer to the referenced literature for such information. Rather than supplying users of the flow chart with such detailed information, its function is intended to be that of a suggestion of considerations to be made, in the form of a holistic overview. While it reflects the processes followed in the development of the NMT, the tool is not built on the first-hand expertise of PD practitioners, but rather on academic literature. A step towards generalisation would be to consider such first-hand knowledge.

The testing tool can be applied to visualise and plan testing efforts for the NMT components and the overall system. It can also be used to communicate these testing efforts among and between teams and people new to the project. Having a holistic overview of the nuances of prototype testing may result in more statistically valid or more useful data. Also, using the tool to communicate the various testing efforts amongst teams could aid in coordinating testing to further enhance the value of obtained results. At this stage, the proposed flow chart has not been applied to plan or communicate testing efforts in the NMT project. Ongoing work aims to test its application in the development of the NMT, which will serve as case study for refining the tool.

Additionally, its generalisation to be used in a variety of PD projects is part of an ongoing qualitative study which seeks to collect knowledge of experts in the field of prototype testing, in order to develop a more universally applicable version of the tool. The presented version is based on existing academic literature, but a lot of expertise about testing in PD remains in the realm of (anecdotal) knowledge of engineering practice ( Tahera *et al.*, 2019). Therefore, we invite readers with expertise in these fields to complement the above-mentioned study by commenting either on this article directly or by contacting the corresponding author.

A generalised, refined version of the flow chart tool could benefit multiple groups of practitioners in PD. For example, test engineers may be able to better design tests and testing rigs with the entire PD process in mind. Project managers, designers, and test engineers can better communicate about tests, timelines, and requirements with a visual aid ( Keller *et al.*, 2006), and designers could develop more appropriate prototypes that are tailored to the planned tests. An important aspect to consider in a generalised version would have to be the use of virtual prototypes, which take an ever-increasingly important role in PD ( Tahera *et al.*, 2015). Given the multitude of PD process models in use ( Wynn & Clarkson, 2018), it is likely that there could not be a single generally applicable flow chart, but rather a modular version that can be adjusted to fit any given company’s processes.

## Conclusion

This paper presents a visual tool to aid in planning and communicating prototype testing efforts for the case of the Nano Membrane Toilet, a complex novel sanitation technology. The tool, a flow chart depicting various aspects of prototype testing throughout the PD process, was developed by collating information about prominently featured aspects of prototype testing in the academic literature.

As the tool is still untested, its validity is not yet confirmed. Ongoing prototype tests that are planned using a previous version of the tool will form the basis of an initial case study. However, an empirical validation of the tool seems unlikely, given the uniqueness of most PD processes. Instead, a qualitative approach collecting and analysing expert knowledge could be a solution toward refinement of the tool.

Much like a product in development, the presented tool will have to undergo more iterations to improve its robustness and its utility. We are currently making steps toward this aim. Nevertheless, we believe that the presented form of the tool, based on a multitude of academic publications, already has value to practitioners in prototype testing and will find use in the development of the NMT.

## Data availability

All data underlying the results are available as part of the article and no additional source data are required.
