# Supplementary material for: Planning and communicating prototype tests for the Nano Membrane Toilet: A critical review and proposed visual tool
Source: Gates Open Res. 2019 Nov 12;3:1532. Originally published 2019 Aug 30. [Version 2] doi: 10.12688/gatesopenres.13057.2 (PMC6974809; doi:10.12688/gatesopenres.13057.2)
Supplement: Peer review history

# Peer review: Reviewer response for version 2

**DOI:** 10.21956/gatesopenres.14233.r28222

**Published:** 2020-01-30

**Authors:** Christer W. Elverum, Aksel Elkjaer

**Affiliations:** 1 Department of Mechanical and Industrial Engineering, Norwegian University of Science and Technology (NTNU), Trondheim, Norway

The authors have taken into account the comments from the first review and made several improvements in the revised version. The changes improve the readability of the paper and present a more coherent theoretical overview. The paper is adequate in its current form, yet there is always room for improvement:

- As commented by another reviewer: the methods section does not describe the method. As it stands now it reads more as a background than a method. 
- The main value of the paper as I see it is the literature review as the case study and the visual overview is too shallow to bring additional value. In this regard, a more elaborate synthesis of literature could have further improved the paper.  

We confirm that we have read this submission and believe that we have an appropriate level of expertise to confirm that it is of an acceptable scientific standard.

# Peer review: Reviewer response for version 2

**DOI:** 10.21956/gatesopenres.14233.r28223

**Published:** 2020-01-21

**Authors:** Khadija Tahera

**Affiliations:** 1 Department of Computing and Engineering, University of Huddersfield, Huddersfield, UK

Thanks for the revised version. Two key issues I wanted to highlight:

- Section Methods explains the case study and the problem but doesn’t explicitly discuss the method of the research. So please give an appropriate title.
- A diagram/schematic of the prototype testing processes is different from a visual test planning tool. I think this paper presents a diagram of the prototype testing processes which is the contribution of the paper. It would be recommended if the authors can clearly mention this and use this consistently.
- Also, as I mentioned before, I think Figure 3 could be elaborated further. For instance, it would be useful to clearly mention how the names of stages were chosen and why these names are different from the ones that are discussed in the literature review.  

I confirm that I have read this submission and believe that I have an appropriate level of expertise to confirm that it is of an acceptable scientific standard.

# Peer review: Reviewer response for version 1

**DOI:** 10.21956/gatesopenres.14186.r27764

**Published:** 2019-10-01

**Authors:** Christer W. Elverum, Aksel Elkjaer

**Affiliations:** 1 Department of Mechanical and Industrial Engineering, Norwegian University of Science and Technology (NTNU), Trondheim, Norway

The paper argues that there is a lack of literature on how to plan and conduct tests, especially for something as complex as the presented case study. The authors then proceed to review four testing approaches and propose a testing strategy that covers the entire product development process.

 The context of the problem is thoroughly covered, including the challenge from the Gates Foundation, sanitation problems and state of the art. The paper covers recent research on testing and addresses testing in product development from a holistic point of view which is an area currently lacking in the literature.

 However, there are some major issues that need to be addressed.

1. Testing approaches:
  - The four testing approaches chosen appear to be quite arbitrarily selected. Why were these chosen over other alternatives?
  - Why is "testing for UCD" presented as a testing approach? This is notably understated as a testing approach as it is a part of a much larger framework. Perhaps "usability testing" is more fitting as a testing approach instead?
  - Is DOE in the same “dimension” as the other approaches? The other approaches have a target (e.g. reliability, compliance, usability) whereas DOE is methodological theory that can be applied when performing a test or experiment. The approaches cannot, in our opinion, be directly compared. DOE certainly has an important role in testing approaches but should perhaps be considered more of a foundational underlying framework than simply an approach.
2. Validity of framework and overall contribution:
  - As the model is untested, even in the context of the NMT, one can question the validity of the model. Most case studies apply an inductive approach where the goal is to create something more generalizable that can be used for other projects as well, this is lacking in this paper. The flowchart simply states which of the four testing methods should be used at each stage, what about focusing on the objectives or outcome as well? In other words, the outcome that is expected/required at each stage to progress the development.
3. Discussion and conclusion should focus more on the applicability to other contexts. How can researchers and practitioners benefit from this work?

In addition to these issues there are some minor issues that should be resolved.

- Additional nuances regarding iterations would be beneficial. Iterations may happen at a smaller scale than highlighted in the paper. Iterations in the development of complex systems do not necessarily happen on a system level. It is assumed the authors have already considered iteration characteristics from the notably different magnitudes of iteration arrows communicating an (undefined?) difference in importance. Do the authors have additional justification for the location, importance and dependencies of the presented iterative loops? Adding information addressing development dependencies to the iteration stages would improve the planning and communication of the visualized strategy. This could be achieved by relating the flowcharts perspective of iterations to agile philosophy advocating the benefits short iterations cycles provide to communication.
- The TRL seems to be forgotten after the introduction. It should perhaps be a part of the visualization as the figure covers most of the development.
- The authors state on multiple occasions that it was necessary to define the test strategy considering the disruptive nature of the technology, but limited focus is given to what impact this caused on the formulation of the flow chart strategy. Justification of which parts of the strategy were directly defined considering a disruptive product innovation may assist in generalizing the proposed framework.
- The NTM is highlighted as an *“unusual case of product development” *due to its combination of highly innovative back-end technology development while maintaining consistent front-end performance. This is comparable to many sustainable product development cases, from electric cars to plastic bags, where the objective is to maintain our current capabilities with new technology. The authors are encouraged to further promote the relevance of the case study in a wider context.

We confirm that we have read this submission and believe that we have an appropriate level of expertise to confirm that it is of an acceptable scientific standard, however we have significant reservations, as outlined above.

# Response

**Published:** 2019-11-08

**Authors:** Jan Hennigs

We want to thank the reviewers' for their comments, which were greatly appreciated and considered in the updated version! Please refer to the 'note to readers' for details of changes in the updated version.

# Peer review: Reviewer response for version 1

**DOI:** 10.21956/gatesopenres.14186.r27767

**Published:** 2019-09-24

**Authors:** Khadija Tahera

**Affiliations:** 1 Department of Computing and Engineering, University of Huddersfield, Huddersfield, UK

- This research is in an interesting topic of prototype testing. This paper has partly addressed the issues of prototype testing and proposed a solution that integrates four approaches/methods/tools however, the rationale of choosing these four approaches is not clearly mentioned.
- A major concern would be that all four of these approaches are considered as testing approaches, which they are clearly not (as my understanding). It needs to clearly come out as to why these approaches are considered as testing approaches.
- The proposed solution has high potential but is in a preliminary stage and would need a significant improvement for scientific publication.
- Please see my detailed comments on the attached document linked here.

I confirm that I have read this submission and believe that I have an appropriate level of expertise to confirm that it is of an acceptable scientific standard, however I have significant reservations, as outlined above.

# Peer review: Reviewer response for version 1

**DOI:** 10.21956/gatesopenres.14186.r27763

**Published:** 2019-09-18

**Authors:** Eckhard Kirchner, Stefan Schork

**Affiliations:** 1 Department of Product Development and Machine Elements, Technical University of Darmstadt, Darmstadt, Germany; 2 Technische Universität Darmstadt, Darmstadt, Germany

In this paper a unified testing strategy flow chart is presented. The flow chart is introduced to support the prototyping process of a nano membrane toilet (NMT) but should be usable in other design projects as well. The strengths of the flow chart are that its application visualizes the planned testing efforts and supports the communication between teams and people new to the project.

 The paper addresses a relevant topic both in engineering and science. It is well structured and the originality of the work is good. There is a high number of relevant and high-quality references and the work of others is acknowledged. The paper is well written and easy to understand but a final proof read to eliminate some minor spelling mistakes is advised.

 Thoughts and recommendations:

1. The paper focuses on the testing of physical prototypes, which is stated in the introduction. However, there is no further explanation of how and why this decision was made. Please elaborate on this decision.
2. The number and quality of references is quite high. Nevertheless, there is a paper by Camburn *et al.* (2017 ^1^) which is missing in my opinion and could provide some useful information.
3. Table 1 shows the technology readiness levels. In this work however, the TRL are only used to explain the current state of development of the NMT (TRL level 6). There is no inclusion of the TRL in the flow chart which raises the question, if the table showing all TRL is needed in this paper. My recommendation is to either use the TRL in the later parts of the paper as well or to delete the table.
4. The flow chart itself is of good quality. There are many “iteration possible” arrows included which raises some questions. Why are there only two of those arrows on the frontend side but six on the backend side? Is there any intention of visualizing which iterations are possible? For example, would an iteration on the frontend side from the validation phase to the exploration phase be possible? My recommendation therefore is to revise the flow chart in terms of visualizing possible iterations and to elaborate those iterations in the text.
5. The conclusion is very short and has more of a summarizing character than a concluding one. Please strengthen this section by pointing out the core aspects of your work.

We confirm that we have read this submission and believe that we have an appropriate level of expertise to confirm that it is of an acceptable scientific standard, however we have significant reservations, as outlined above.

## References

[1] : Design prototyping methods: state of the art in strategies, techniques, and guidelines. *Design Science*.2017;3: 10.1017/dsj.2017.10 10.1017/dsj.2017.10
